# Hi-C analysis: from data generation to integration

**DOI:** 10.1007/s12551-018-0489-1

**Published:** 2018-12-20

**Authors:** Koustav Pal, Mattia Forcato, Francesco Ferrari

**Affiliations:** 1 IFOM, the FIRC Institute of Molecular Oncology, Milan, Italy; 2 Department of Life Sciences, University of Modena and Reggio Emilia, Modena, Italy; 3 Institute of Molecular Genetics, National Research Council, Pavia, Italy

**Keywords:** Chromatin 3D architecture, Epigenomics, Computational biology, High-throughput sequencing, Chromosome conformation capture

## Abstract

In the epigenetics field, large-scale functional genomics datasets of ever-increasing size and complexity have been produced using experimental techniques based on high-throughput sequencing. In particular, the study of the 3D organization of chromatin has raised increasing interest, thanks to the development of advanced experimental techniques. In this context, Hi-C has been widely adopted as a high-throughput method to measure pairwise contacts between virtually any pair of genomic loci, thus yielding unprecedented challenges for analyzing and handling the resulting complex datasets. In this review, we focus on the increasing complexity of available Hi-C datasets, which parallels the adoption of novel protocol variants. We also review the complexity of the multiple data analysis steps required to preprocess Hi-C sequencing reads and extract biologically meaningful information. Finally, we discuss solutions for handling and visualizing such large genomics datasets.

The total length of DNA contained in a human cell would be 2 m long if completely stretched, i.e., considering the cumulative size of 6 billion nucleotides composing a diploid genome. However, such a long polymer must fit into a nucleus with an average diameter of 10 μm, i.e., five orders of magnitude shorter than the genome (Marti-Renom and Mirny 2011). This is not only a structural challenge, but also a functional one, as the genome must be densely packed, while at the same time preserving its function, i.e., being accessible to factors regulating transcription and replication. This is achieved thanks to the fact that the DNA inside the cell is never naked, but always associated to many proteins with a structural and functional role. The complex of DNA and associated proteins is named chromatin and its 3D organization inside the nucleus is not random but tightly regulated (Cavalli and Misteli 2013).

Our knowledge of chromatin 3D organization has greatly increased over the past 20 years thanks to the development of novel experimental techniques, including high-resolution and high-throughput imaging techniques (Huang et al. 2010; Zane et al. 2017) and other molecular biology techniques. Among the latter, chromosome conformation capture (3C) (Dekker et al. 2002) and its high-throughput derivatives have been the most prominent ones. 3C allows probing physical interaction between non-adjacent genomic loci. The technique is based on cross-linking of DNA and associated proteins to stabilize chromatin 3D structure, then digesting DNA with restriction enzymes. The loose DNA fragment ends are then re-ligated, so as to obtain hybrid molecules, which may contain two fragments of DNA that were not adjacent but indeed far apart in the original linear genomic sequence. The fact that they are ligated together at the end of the process indicates some degree of physical proximity at the beginning of the experimental procedure. By analyzing the resulting hybrid molecules, we can assess the physical interaction between distant genomic loci (Belton et al. 2012). This can be assessed with PCR, using a pair of primers specifically designed to target predefined regions, as per the original 3C protocol. However, other high-throughput derivatives of 3C based on microarrays hybridization (Dostie et al. 2006; Simonis et al. 2006) or high-throughput sequencing have been proposed subsequently. Among them, 4C allows detecting pairwise interactions between one target anchor point and potentially any other genomic region (van de Werken et al. 2012), whereas 5C allows probing multiple pairwise interactions between predesigned anchor points (Phillips-Cremins et al. 2013). Hi-C is the most comprehensive and high-throughput derivative, allowing us to score contact frequency between virtually any pair of genomic loci (Lieberman-Aiden et al. 2009). This results in very large and complex datasets, especially for large genomes, as the number of possible pairwise interactions increases exponentially with the genome length. As such in this review on big-data challenges in epigenomics, we will focus especially on datasets obtained from mammalian genomes, as well as on data analysis solutions used in this context.

## Hi-C data availability: increasing size and resolution

Hi-C data allows examining the genome 3D organization at multiple scales (Rocha et al. 2015; Fraser et al. 2015). On a large scale, the genome is organized in distinct “compartments.” Namely, active (“A”) and inactive (“B”) compartments have been identified from Hi-C contact maps analysis, and they correlate with the presence of active or inactive chromatin domains, respectively. The active compartment includes genomic regions characterized by transcription or epigenetic marks associated to open chromatin. Instead the inactive compartment covers regions with compact heterochromatin and gene expression silencing epigenetic marks (Lieberman-Aiden et al. 2009). When analyzing local patterns in the contact matrix instead, the topologically associating domains (TADs) emerge as a key feature, i.e., regions characterized by high intradomain contact frequency, and reduced interdomain contacts (Sexton et al. 2012; Dixon et al. 2012; Nora et al. 2012). On an even finer scale, Hi-C data have been used to identify specific points of contact between distant chromatin regions. Sometimes interactions are called chromatin loops, when referring to intrachromosomal (*cis*) contacts (Jin et al. 2013; Rao et al. 2014). This level of analysis is especially challenging for the resolution limit of Hi-C data.

Hi-C data resolution is primarily defined by (1) the restriction enzymes used in the experimental procedure and by (2) the sequencing depth. Over the years, we have witnessed an attempt to increase the resolution of Hi-C data by working on these parameters, resulting in available datasets characterized by increasing size and resolution, reaching very high numbers of sequenced reads, especially for mammalian genomes. In addition, specific protocol variations have been proposed with the aim of improving the resolution.

The classical Hi-C technique involves restriction digestion of a formaldehyde cross-linked genome with sequence specific restriction enzymes, followed by fill in and repair of digested ends with the incorporation of biotin-linked nucleotides. The repaired ends are then re-ligated. Finally, the cross-linking is reversed and associated proteins are degraded. This produces the ligation products which are then nonspecifically sheared, generally by sonication, and enriched for sheared fragments containing the ligation junction, using a biotin pull-down strategy, and finally sequenced using paired-end sequencing (Belton et al. 2012). The enrichment step aims to select sonicated fragments containing the ligation junction, increasing the proportion of informative non-same fragment read pairs (mate pairs originated from different restriction fragments).

Among protocol variations aimed at increasing resolution, the widely adopted in situ Hi-C achieves cleaner and stronger signal by performing all the protocol steps up to ligation in intact cell nuclei (Rao et al. 2014). This reduces spurious ligation events as well as dangling ends reads, i.e., read pairs originating from non-ligated fragments, thus de facto increasing the number of usable reads. A very different approach, yet having similar effects on resulting reads quality, was the tethered conformation capture (TTC), based on performing ligations with cross-linked DNA fragments attached to a solid substrate, rather than in solution (Kalhor et al. 2011). More recently, Hi-C 2.0 has been proposed as a protocol variant to reduce spurious ligation events in the mixture of sequenced molecules (Belaghzal et al. 2017). Hi-C 2.0 takes into account recent advances in the field, such as the removal of SDS solubilization step after digestion, first used in 4C (Splinter et al. 2012), then in single-cell Hi-C (Nagano et al. 2013) and finally adapted in in situ Hi-C (Rao et al. 2014). It also incorporates the advances made in other studies by using frequent cutting restriction enzymes such as micrococcal nuclease (Hsieh et al. 2015) and MboI (Rao et al. 2014).

In this context, it’s worth mentioning also the capture Hi-C protocol variants, which achieve higher resolution over specific target regions by directly enriching for ligation products involving a selected set of target sequences. For example, promoter capture Hi-C is designed to enrich for interactions centered around a selected set of annotated promoters (Schoenfelder et al. 2015; Mifsud et al. 2015). In this context, more recently Bridge Linker-Hi-C (BL-Hi-C) was proposed as a solution to favor the detection of interactions mediated by structural or regulatory proteins. BL-Hi-C uses a two-step ligation with an intervening linker to enrich for ligation products originating from DNA fragments connected by a cross-linked protein (Liang et al. 2017).

The ultimate resolution limit of Hi-C data is the restriction fragment resulting from DNA digestion. The original Hi-C protocol was based on HindIII and NcoI restriction enzymes, both recognizing and cutting a 6 bp long sequence: AAGCTT and CCATGG, respectively (Lieberman-Aiden et al. 2009). Later publications also adopted 4 bp cutters such as Dpn-II (Sexton et al. 2012; Rao et al. 2014), which has more abundant target restriction sites (GATC sequence), thus resulting in smaller fragments, so as to increase the resolution. More recently, alternative protocol variations have been introduced to leverage even shorter restriction fragments. Namely, the COLA protocol makes use of a restriction enzyme recognizing an RCGY motif (with R equal to A or G and Y equal to C or G) to achieve an even smaller average fragment size, thereby allowing experimentalists to probe complex chromatin conformations which may involve three or more interacting genomic loci (Darrow et al. 2016). Other protocol variations aimed at increasing Hi-C resolution include in situ DNase Hi-C, which replaces restriction enzymes with the endonuclease DNase I (Ramani et al. 2016), as well as Micro-C, which uses micrococcal nuclease to obtain single nucleosome scale chromatin conformation maps (Hsieh et al. 2015).

However, the most striking effort in improving Hi-C data resolution has been focused on increasing the sequencing depth (Table 1). In the 9 years following the first study, articles adopting this technique have achieved new records in terms of total number of reads sequenced. While the first Hi-C dataset had 95 million reads in total (up to 30 million per sample), the most recent articles have reached up to 40 billion reads per dataset (up to 7.3 billion per sample) (Belaghzal et al. 2017; Rowley et al. 2017; Bonev et al. 2017). The increase in sequencing depth has been paralleled by a decrease in the size of genomic bins used to summarize Hi-C signal. The developers of in situ Hi-C have been the first ones to reach 1 kb resolution in a human genome Hi-C map, binned at 950 bp (Rao et al. 2014), and more recently Bonev et al. (Bonev et al. 2017) reached an even higher coverage on a mouse genome dataset. It’s worth remarking that when applied to smaller genomes (e.g., Drosophila), a smaller amount of reads can yield higher coverage, so that higher resolution analysis is possible. The first Hi-C dataset in Drosophila, based on the simplified Hi-C protocol which lacks biotin incorporation and enrichment, already allowed to examine contact maps at 40 kb resolution and to clearly highlight topological domains (Sexton et al. 2012). More recently, the local chromatin topology of the Drosophila genome has been investigated at a resolution of 500 bp (average fragment size) to characterize domains at sub-kb resolution (Wang et al. 2018a).

**Table 1:** Hi-C studies over the past decade that marked forward leaps in resolution or dataset size

Study	Organism	Restriction enzyme				Hi-C protocol	Read pairs	Max. binning res.
		6 bp		4 bp				
		HindIII	NcoI	DpnII	MboI			
Lieberman-Aiden et al. 2009	Human	✓	✓			Dilution	30M	1 mb
Sexton et al. 2012	Drosophila			✓		Simplified	362.7M	10 kb
Dixon et al. 2012	Human, mouse	✓				Dilution	806.1M	40 kb
Jin et al. 2013	Human	✓				Dilution	2.9B	5 kb
Rao et al. 2014	Human, mouse			✓	✓	In situ	6.5B	950 bp
Rao et al. 2017	Human				✓	In situ	6.8B	5 kb
Bonev et al. 2017	Mouse			✓		In situ	7.3B	850 bp
Wang et al. 2018a	Drosophila			✓		In situ	1.5B	frag.

The table reports the original publication (study), organisms examined, restriction enzymes used, protocol variation, maximum number of read pairs sequenced per sample, and maximum binning resolution used in the analyses presented by the original authors. The size of restriction sites (6 bp or 4 bp) is also indicated

*M* is for million read pairs, *B* is for billion read pairs, *frag.* is for fragment level analysis

The increase in complexity of individual datasets has been paralleled by a global increase in publicly available chromatin architecture data. It’s especially worth mentioning the resources, in term of available datasets, provided by large-scale consortia including ENCODE (Davis et al. 2018), covering multiple cell lines, the Roadmap epigenomics (Dixon et al. 2015), and related efforts (Schmitt et al. 2016a), with data from several primary tissues and cultured cells, and the recently established 4D Nucleome consortium (Dekker et al. 2017) which is committed to release a number of datasets covering multiple cell types, conditions, and treatments. As more and more datasets become available, it will become increasingly important to establish common and standardized procedures to assess data quality (Wolff et al. 2018) and reproducibility of replicates (Yardimci et al. 2017; Yang et al. 2017).

## Hi-C data analysis: from FASTQ to interaction maps

Hi-C data analysis is a process involving multiple steps that can be separated in preprocessing, i.e., from raw data to the Hi-C contact matrix, and downstream analyses (Ay et al. 2014; Schmitt et al. 2016b).

**Fig. 1:**
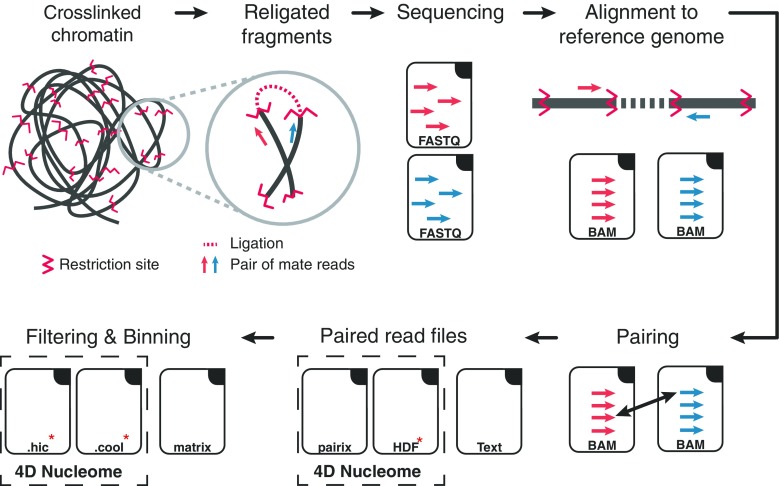
Hi-C data, from generation to contact matrix. The figure shows a schematic representation of Hi-C data analysis, starting from a cartoon depicting cross-linked chromatin and a prototypic pair of mate reads positioned on the restriction fragments from which they originate. Raw sequencing paired-end reads (in FASTQ files) are aligned to the reference genome considering the mate reads independently. Then, aligned reads (in BAM files) are assigned to their fragment of origin and paired. The paired reads are stored in a sorted file that can be in either plain text, indexed text (pairix), or binary (e.g., HDF) formats, depending on the pipeline. Finally, after filtering and binning, the read counts are stored in contact matrix files, including plain text (e.g., 2D or sparse matrix) or binary (e.g., hic or cool) file formats

Preprocessing starts with FASTQ files of paired-end reads obtained from high-throughput sequencing that are (1) first of all aligned to the reference genome, (2) then filtered to remove spurious signal, and then read counts are (3) binned and (4) normalized. The last two steps are often performed simultaneously but involve distinct choices that affect the characteristics of the normalized contact matrix obtained as final output (Fig. 1).

Hi-C paired-end reads are aligned separately, as they are expected to map in different unrelated regions of the genome due to the peculiarity of these data. The alignment can be performed with standard tools such as bowtie (Langmead and Salzberg 2012) or bwa (Li and Durbin 2009; Dixon et al. 2012) aiming to align the full length read, as done especially in the earlier analysis pipelines (Yaffe and Tanay 2011). However, the alignment of Hi-C reads may prove challenging in case the read spans the ligation junction, thus having two portions of the read itself matching distinct genomic positions. These are also termed “chimeric reads” and their alignment requires specific strategies to attempt mapping different portions of the read, which is expected to yield a higher fraction of mappable reads, especially when the reads are longer (Forcato et al. 2017). Several variations of chimeric reads mapping approaches are now implemented in many pipelines including ICE (Imakaev et al. 2012), TADbit (Serra et al. 2017), HiCUP (Wingett et al. 2015), HIPPIE (Hwang et al. 2015), Juicer (Durand et al. 2016b), and HiC-Pro (Servant et al. 2015).

Aligned reads are then filtered to remove spurious signal due to experimental artifacts. While reads filtering is an analysis part common to many high-throughput sequencing applications, it is particularly important for Hi-C data as multiple steps in the experimental protocol can generate biases in the sequencing results. Read level filters include the removal of reads with low alignment quality or PCR artifacts, i.e., multiple read pairs mapped in the same positions. Then, read pairs filters are based on the distance of aligned reads to the downstream restriction site, which is used to estimate if the read pair is compatible with the expected size of sequenced fragment obtained from the ligation product (Yaffe and Tanay 2011). Moreover, read pairs can be filtered if they are mapped on the same fragment, thus resulting from lack of ligation or self-ligation events, or if their orientation and distance in mapping positions is compatible with an undigested chromatin fragment (Jin et al. 2013). More recently, the MAD-max (maximum allowed median absolute deviation) filter on genomic coverage has been proposed to remove low-coverage bins, identified as bins that are three standard deviations below the center of a log-normal distribution which fits the total number of contacts per genomic bin (Nora et al. 2017; Schwarzer et al. 2017). Hi-C protocol variants yielding a cleaner signal result in less spurious reads, thus being less affected by the filters described here (Forcato et al. 2017).

Although the reads are mapped and counted on individual restriction fragment ends, Hi-C data are usually not analyzed at single-fragment level. Instead, the read counts are generally summarized at the level of genomic bins, i.e., a continuous partitioning of the genome in intervals of fixed size. The rationale behind this approach is that genomic bins allow achieving a more robust and less noisy signal in the estimation of contact frequencies, at the expense of resolution. While the restriction fragment remains the ultimate physical limit to Hi-C resolution, the choice of the bin size used to summarize results is de facto defining the final resolution of analysis results. Some practical strategies have been specifically proposed to support pushing the limit of bin size choice to smaller and smaller bins, such as having at least 80% of bins covered by 1000 reads (Rao et al. 2014). Recently, two approaches to determine optimal bin size have been proposed: in deDoc, the bin size is selected as the one at which the structural entropy of the Hi-C matrix reaches a stable minimum (Li et al. 2018), whereas QuASAR requires the presence of replicates and compares quality and replicate scores of the samples to find the maximum usable resolution (Sauria and Taylor 2017). Some publications also attempted to score interaction frequencies using single-fragment level data both in human (Jin et al. 2013) and smaller genomes, such as Drosophila (Ramírez et al. 2015), where a relatively higher coverage can be achieved with a lower number of reads. From an alternative point of view, HiCPlus attempts to enhance resolution of shallowly sequenced datasets by applying deep convolutional neural network; the authors showed that using only 1/16 of the original reads, they can impute matrices similar to the original ones (Zhang et al. 2018).

The final preprocessing step is normalization. Read counts binning and normalization are usually coupled and performed simultaneously by the same tools. Hi-C normalization strategies can be divided in two main groups: explicit and implicit (or matrix-balancing) normalization methods. The explicit normalization methods are based on the explicit definition of a set of biases known to be associated to Hi-C reads or high-throughput sequencing in general, thus affecting the resulting read counts per restriction fragment. These include the fragment length, its GC content and mappability. Correction factors are computed for each of the considered biases and their combination, then applied to the read counts per genomic bin (Yaffe and Tanay 2011; Hu et al. 2012; Jin et al. 2013). The implicit or matrix-balancing normalization methods instead do not rely on any specific assumptions on the sources of biases in Hi-C read counts. They are instead based on the assumption that each genomic locus should have “equal visibility,” i.e., the interaction signal, as measured by Hi-C for each genomic locus, should add up to the same total amount. These include the sequential component normalization (SCN) (Cournac et al. 2012), the iterative correction and eigenvector decomposition (ICE) normalization (Imakaev et al. 2012), and Knight-Ruiz matrix-balancing approach (Knight and Ruiz 2013; Rao et al. 2014), implemented by multiple tools (Sauria et al. 2015; Servant et al. 2015; Durand et al. 2016b; Kumar et al. 2017; Wolff et al. 2018; Stansfield et al. 2018). ICE normalization has also been optimized for handling large- and high-resolution datasets (Kerpedjiev et al. 2018).

A still open problem is the normalization of Hi-C data originating from genomes with copy number alterations. While matrix-balancing approaches should partially cancel out the unbalances in total Hi-C signal originating from any locus, the resulting local distortions in the interaction matrix are not completely corrected. An earlier work proposed a solution with an additional scaling factor to be applied on top of ICE normalization to correct for aneuploidies with whole chromosome duplications or deletions (Wu and Michor 2016). Recent publications proposed instead more generalizable solutions adding a correction factor to matrix-balancing normalization to model and adjust the effect of local copy number variations (Vidal et al. 2018; Servant et al. 2018).

## Downstream analyses

Include all the methods used to extract biologically meaningful results from Hi-C data matrices at multiple levels of resolution, including (1) the identification of compartments, (2) calling TADs, and (3) point of interactions, also termed loop calling when referred to *cis-*interactions (Forcato et al. 2017) (Table 2).

**Table 2:** List of tools for downstream analyses on Hi-C data

Method	Compartment	TAD	Interaction	Visualization	Input format		Reference
					Text	Binary	
CScoreTool	✓				RP		Zheng and Zheng 2018
HiTC	✓			✓	SM		Servant et al. 2012
HOMER	✓	✓	✓		RP		Heinz et al. 2010
Juicer (HiCCUPS, Arrowhead, Juicebox)	✓	✓	✓	✓		hic	Durand et al. 2016a, b
4D NAT		✓		✓	2D,SM	HDF	Seaman and Rajapakse 2018
3DNetMod		✓			SM		Norton et al. 2018
Armatus		✓			2D		Filippova et al. 2014
CaTCH_R		✓			SM		Zhan et al. 2017
ClusterTAD		✓			2D		Oluwadare and Cheng 2017
domainCaller		✓			2D		Dixon et al. 2012
deDoc		✓			2D		Li et al. 2018
HiCDB		✓			2D		Chen et al. 2018
HiTAD		✓			SM		Wang et al. 2017
HiCseg		✓			2D		Lévy-Leduc et al. 2014
IC-Finder		✓			2D		Haddad et al. 2017
InsulationScore		✓			2D		Crane et al. 2015
Lavaburst		✓			2D		Schwarzer et al. 2017
MrTADFinder		✓			SM		Yan et al. 2017
Matryoshka		✓			2D		Malik and Patro 2018
TADBit		✓			2D		Serra et al. 2017
TADTree		✓			2D		Weinreb and Raphael 2015
TADtool		✓		✓	2D,SM	npy	Kruse et al. 2016
CHiCAGO			✓			BAM	Cairns et al. 2016
diffHiC	✓		✓			HDF	Lun and Smyth 2015
FastHiC			✓		SM		Xu et al. 2016a, b
Fit-Hi-C			✓		SM		Ay et al. 2014
GOTHiC			✓		RP		Mifsud et al.*,*^2017^
HIPPIE			✓				Hwang et al. 2015
PSYCHIC			✓		2D		Ron et al. 2017
3D Genome Browser				✓	RP,2D,SM	BUTLR	Wang et al. 2018b
HiCExplorer		✓		✓	RP		Ramírez et al. 2015
HiGlass				✓		HDF	Kerpedjiev et al. 2018
gcMapExplorer				✓		HDF	Kumar et al. 2017
WashU Epigenome Browser				✓		hic, HDF	Zhou et al. 2013

The table report the list of methods with their reference name, the capability of each tool in term of calling compartments, TADs, interactions, or for visualizing data. Tools are grouped based on their main focus in term of analysis type (compartments, TADs, interactions, calling, and visualization), and within each group are sorted alphabetically by tool name. The format of input data is reported, by specifying if the tools accepts text or binary input file formats. The last column reports the reference publication for each tool

Read pairs (RP), 2D matrix (2D), sparse matrix (SM) and python numpy matrix (npy) file formats

### Tools to call compartments

Compartments are the first level of chromatin organization which was derived from the analysis of Hi-C data in (Lieberman-Aiden et al. 2009). They clearly emerged in the Hi-C map as a plaid pattern after calculating Pearson correlation of the distance normalized map. To define active (“A”) and inactive (“B”) compartments, the authors used the sign of the first eigenvector (first principal component). This widely used approach is implemented in multiple tools, with small differences regarding the way the matrix is normalized before Pearson correlation calculation and PCA analysis. The original approach calculated the correlation of a matrix of observed over expected Hi-C signal ratio, where the expected signal was obtained from a distance normalized contact matrix. A similar approach is available in HOMER (Heinz et al. 2010), whereas loess calculation of distance dependency is implemented in Cworld (https://github.com/dekkerlab/cworld-dekker) and in the HiTC R package (Servant et al. 2012). The eigenvector module of Juicer allows using alternative observed matrixes (raw or balanced) (Durand et al. 2016b). CscoreTool (Zheng and Zheng 2018) instead is not based on PCA to call compartments, but relies on a faster and memory efficient approach defining a compartment score reflecting the chance of any given bin to be in the “A” compartment. A detailed guide for the identification and annotation of compartments is reported in (Miura et al. 2018).

### TAD callers

As with compartments, TADs were first identified by visual inspection of the interaction maps. Here, they appear along the diagonal of the contact matrix as blocks of highly self-interacting regions. This observed pattern guided the design of TAD-calling algorithms. Only subsequently to their observation, their biological properties, putative function and genesis were investigated, reporting, e.g., the enrichment of insulator proteins binding at TAD boundaries. More recently, genetic perturbation experiments have been clarifying how TADs are formed and what is their relationship with other structures observed in the genome (Rao et al. 2017; Nuebler et al. 2018). However, an unambiguous definition of TADs is still evolving. As a consequence, even if apparently evident on the matrix, their computational identification is not straightforward yet (Dali and Blanchette 2017). The biggest challenge to a rigorous methods benchmarking is probably the lack of a set of true, experimentally validated TADs. Simulated data are also problematic as they lack the complexity of real data. Yet, several methods have been proposed, assessing their performance on metrics such as the reproducibility of results among replicates, enrichment in insulators at domain boundaries, or comparing to the first genome-wide identification of TADs by (Dixon et al. 2012). An important aspect to review TAD callers is how they deal with data resolution. This comprises the ability of defining domains combining results obtained at different resolutions, but also to the ability of being computationally efficient on high-resolution datasets or being able to identify TADs even in sparse matrices.

The first methods developed to call TADs were based on one-dimensional scores. The directionality index (DI) calculates for each bin the degree of upstream and downstream interaction biases and is segmented with a hidden Markov model (HMM) to derive TADs in DomainCaller (Dixon et al. 2012) and the 4D Nucleome Analysis Toolbox (Seaman and Rajapakse 2018). Instead, the insulation score quantifies the interactions passing across each genomic bin, and it allows defining boundaries by identifying local minima (Crane et al. 2015), also implemented in Cworld (https://github.com/dekkerlab/cworld-dekker). Other methods are not based on a one-dimensional score, but aim to identify the best partitioning of the contact matrix in TADs based on clustering algorithms, such as HiCseg or ClusterTAD (Lévy-Leduc et al. 2014; Oluwadare and Cheng 2017), or other partitioning algorithms, such as TADBit (Serra et al. 2017).

These methods identify only one level of TADs, but the increased resolution available in newer datasets highlighted the existence of a hierarchical structure of TADs inside other TADs. Many tools are now explicitly addressing this with multiscale analysis approaches.

The idea of calculating domains at different resolutions was first introduced by Armatus (Filippova et al. 2014). Armatus identifies resolution specific domains and calculates a consensus set of domains conserved across resolutions. It formulates the problem of TAD calling as the optimization of a scoring function based on their local density of interactions and features a tuneable parameter (gamma) that correlates with resolution. Other methods implemented variations of this approach with different objective functions, with the aim of achieving better computing performance to work on higher resolution datasets and of facilitating the tuning of parameters required to the user. Matryoshka (Malik and Patro 2018) uses a variant of Armatus to extract domains at different scales and then predicts the hierarchy by clustering the domains based on the variation of information distance. MrTADFinder (Yan et al. 2017), Lavaburst (Schwarzer et al. 2017), and 3DNetMod (Norton et al. 2018) borrow concepts from graph theory: by representing Hi-C maps as graphs, the identification of TADs is treated as a community detection problem in a network. Here, the objective function is a modularity score and sweeping over a range of gamma parameters allows generating a hierarchy of TADs. An approach exploiting a different property of graphs is deDoc (Li et al. 2018), which minimizes the structural entropy (i.e., the global uncertainty) of the Hi-C map. It is designed to work well with sparse matrices and proposes structural entropy as a mean to identify the proper bin size for the dataset under investigation. Other multiscale methods able to examine the hierarchy of TADs include Arrowhead (Rao et al. 2014; Durand et al. 2016b), TADtree (Weinreb and Raphael 2015), IC-Finder (Haddad et al. 2017), CaTCH (Zhan et al. 2017), and HiTAD (Wang et al. 2017). HiCDB (Chen et al. 2018) detects TAD boundaries based on local relative insulation, a variation of the insulation score approach that calculates insulation using different windows sizes and incorporating information from the local background. Finally, TADtool integrates interactive data exploration functionalities to directly select parameters for TAD calling based on DI and insulation score (Kruse et al. 2016).

Among such a large production of TAD calling methods, it’s worth mentioning localtadsim, a recently published approach to quantitatively compare multiple topological domains definitions (Sauerwald and Kingsford 2018).

### Interaction callers

Interactions are specific points of contact between distant chromatin regions, such as those occurring between promoters and enhancers. The computational identification of interactions requires the definition of a background model in order to discern contacts with an interaction frequency higher than expected. The background can be estimated using local signal distribution or modeled using global (chromosome-wide or genome-wide) approaches. Fit-Hi-C (Ay et al. 2014) uses nonparametric splines to estimate the global background distribution from the data. Other methods defining a global background model are GOTHiC (Mifsud et al. 2017), HIPPIE (Hwang et al. 2015), and HOMER (Heinz et al. 2010). HiCCUPS (Rao et al. 2014) and diffHic (Lun and Smyth 2015) are instead based on a local enrichment score, comparing the signal of each bin pair against its neighborhood. HiCCUPS is implemented in the Juicer pipeline (Durand et al. 2016b) and is a popular method to identify reliable chromatin loops in high-resolution datasets. However, even in high-resolution datasets, it returns only a few thousand loops (e.g., 9448 interactions from a 5 kb resolution GM12878 Hi-C map were reported by (Rao et al. 2014)), which are useful to study the general structure of chromatin (Rao et al. 2017) but cannot provide a comprehensive picture of all the interactions between promoters and regulatory elements, as not all of the identified interactions feature a promoter. Moreover, HiCCUPS was designed for high-resolution datasets, on which yields better performances (Forcato et al. 2017), and the authors recommend its application only on Hi-C maps with more than 300 million contacts.

Another local enrichment approach is implemented in PSYCHIC (Ron et al. 2017), a solution explicitly taking into account the TAD structure to identify significant interactions against a TAD-specific background model. The genome is segmented into domains that are merged to define a TAD hierarchy. Then, for each TAD, interactions are modeled according to a piecewise power law regression. As a comparison, in the same sample of the (Rao et al. 2014) dataset described above, PSYCHIC identified about 30,000 interactions involving promoters.

Finally, FastHiC (Xu et al. 2016b) differentiates from the other methods because it explicitly models the spatial dependency among adjacent loci, considering the fact that interaction frequencies of pairs of neighboring loci may be correlated. It is a computationally more efficient reimplementation of the HMRFBayesHiC (Xu et al. 2016a) method designed for high-resolution data.

Even if Hi-C allows identification of any type of chromatin interaction, these interactions happen between genomic bins of several kb, and the maximum resolution achieved in mammalian genomes is about 1 kb, at the cost of sequencing billions of reads. When the interest is limited to interactions between promoters and regulatory elements or between specific loci (e.g., SNPs), Capture-HiC (cHi-C) is a more advisable technique (Hughes et al. 2014; Mifsud et al. 2015). Although the preprocessing steps (alignment, filtering) can be conducted on cHi-C data with the same methods designed for Hi-C (e.g., HiC-Pro (Servant et al. 2015), HiCUP (Wingett et al. 2015)), caution must be taken when trying to identify significant interactions. Differently from Hi-C, capture Hi-C is an asymmetric assay capturing “many vs. all” interactions; it is affected by experimental biases due to differential capture efficiency; and it is often processed at fragment level resolution. CHiCAGO (Cairns et al. 2016) addresses these problems by using a combined background distribution (negative binomial and Poisson), a specific implicit normalization and using an approach for multiple tests correction based on *p* value weighting, to adapt the stringency of the test to the genomic distance of the tested interaction. CHiCAGO identified 88,667 promoter interactions in a cHi-C experiment of the GM12878 cell line.

More recently, Hi-C datasets with very high coverage allowed adopting ad hoc solutions independent of a precomputed bins strategy, also termed bin-free or bin-less analysis approaches (Cohen et al. 2017; Spill et al. 2017). These methods perform normalization and interactions calling without relying on predefined genomic bins to partition the interaction matrix, but apply instead different strategies to locally identify the best range of distances to aggregate read counts. In particular, SHAMAN has been already applied to the study of Drosophila genome activation during early stages of embryo development (Ogiyama et al. 2018) and to the analysis of the mouse genome to investigate the relationship between transcription and insulation, during neural stem cells differentiation (Bonev et al. 2017).

## Handling Hi-C data—data formats and tools for high-resolution matrices

The lack of a common standard in data formats has already been reported as a critical issue in the field of Hi-C data analysis and its definition is one of the goals of the 4D Nucleome project (Dekker et al. 2017). Most tools presented in this review store data in different formats, and only few provide utilities to convert from one format to another. This hampers the possibility for a not expert user to test multiple computational approaches and define a preferred pipeline.

FASTQ and BAM are the current standard for sequenced and aligned reads, respectively. When it comes to the creation of the read pairs (pairs file) and the interaction map files, these are saved in formats that vary greatly among the tools (Fig. 1). The basic information to be saved in pairs file is the genomic location of the aligned mate reads (chromosome, start, strand), but other fields contained in the BAM files, such as read names, alignment quality, and cigar strings are sometimes reported as well. The 4D Nucleome recently proposed “pairix,” an indexed text file format derived from “tabix” to save pairs (https://github.com/4dn-dcic/pairix/blob/master/pairs_format_specification.md).

The most intuitive way to save the Hi-C map is as a plain text symmetric matrix, where the first column and first row contain the bin identifiers. HOMER (Heinz et al. 2010) adopts a similar format, also called “dense” format. Since Hi-C matrices are symmetric and sparse, a more efficient format is the “sparse” format where only nonzero entries of half of the matrix are reported as “row column value” triplets. This format is also called coordinated list or COO and is used by HiC-Pro (Servant et al. 2015). In both formats, the bin IDs can be replaced by their chromosomal coordinates; otherwise, another file with the position of each bin is normally provided. In the case of high-resolution matrices, using these formats can produce files that are large and difficult to manage. To overcome this problem, matrices can be saved using highly compressed binary formats: the “.cool” format is based on HDF5 and is used by the cooler pipeline (Kerpedjiev et al. 2018); the “.hic” format is used instead by the Juicer pipeline (Durand et al. 2016b). Both these formats are being used by the 4D Nucleome consortium to disseminate their datasets.

Other developed formats are an indexed binary file format called Binary Upper TrianguLar matRix (BUTLR) used for visualization by the 3D Genome Browser (Wang et al. 2018b) and the genome contact map format (“gcmap”) based on HDF5 and used for analysis and visualization by the Genome contact map explorer (Kumar et al. 2017). Finally, an attempt to create a suite of tools for formats conversion, manipulation, and 2D genomic arithmetic of Hi-C data (similar to bedtools) is pgltools which is based on the paired-genomic-loci data (PGL) format (Greenwald et al. 2017). Converting between these formats is not always straightforward and may require several steps (see examples in Miura et al. (2018)). For example, Juicer provides utilities to convert “.hic” files into sparse matrices, but to convert sparse or dense matrices into “.hic” files an intermediate text format is required. Using some of these formats (hic, cool, gcmap), it is possible to save the same matrix binned at various resolutions in a single file, which is convenient for visualization purposes.

## Handling Hi-C data—data visualization tools

The visualization of Hi-C data is a crucial part of Hi-C data analysis (see also Yardimci et al. (2017)). Thanks to visual inspection of the Hi-C matrices, compartments and TADs have been discovered, and new patterns have been observed and described, e.g., stripes in (Vian et al. 2018). Moreover, the thousands of features resulting from downstream analyses are more easily summarized and interpreted by visual representation, overlaid to the Hi-C contact matrix. This poses some challenges due to the two-dimensional nature and the size of this kind of data.

Several tools address these issues and display Hi-C contact maps as heatmaps, supporting matrices saved in binary formats to allow fast retrieval of the data. Juicebox (Durand et al. 2016a), gcMapExplorer (Kumar et al. 2017), and HiGlass (Kerpedjiev et al. 2018) allow to smoothly browse Hi-C heatmaps interactively, to zoom in and out with different resolutions, to visualize maps together with other genomics data such as ChIP-seq and to compare multiple maps in a synchronous way. Juicebox is available both as a desktop and a cloud-based web application named Juicebox.js (Robinson et al. 2018). It loads matrices in “.hic” format and its strengths are its intuitive interface and easy use. gcMapExplorer is a Python software featuring a GUI that loads data in the “.gcmap” format; it also performs different types of normalizations on raw matrices. HiGlass is available as a docker container and loads matrices in “.cool” format. It allows sophisticated customization of the layout by juxtaposing panels with multiple maps at the desired zoom levels, along with other genomic data. Juicebox and HiGlass allow sharing a session via a URL or a JSON representation, respectively, which can also be easily hosted at web sites.

Other tools such as WashU Epigenome Browser (Zhou et al. 2013) and the 3D Genome Browser (Wang et al. 2018b) adopt a more classical genome browser configuration, where the heatmap is rotated by 45° and displayed as a triangle, with the diagonal aligned horizontally to other genomic tracks. This type of representation is useful to display chromatin conformation at selected loci. WashU Epigenome Browser is able to load both “.hic” and “.cool” formats, whereas the 3D Genome Browser supports the “.butlr” format but allows visualizing only one resolution at a time. This type of visualization is also supported by HiGlass.

Finally, HiCExplorer instead is a more complex framework available as command line tools or Galaxy module for a web interface (Wolff et al. 2018). In addition to data visualization functions, HiCExplorer includes also command to perform analyses, such as calling TADs.

## Conclusion

Overall, the rapid widespread adoption of Hi-C and its variants have spurred an explosive growth of complexity and size in available chromatin 3D architecture datasets. This, coupled with the rapid flourishing of many data analysis approaches, has already raised substantial concerns in the field about the need for common standards and guidelines (Marti-Renom et al. 2018). This is even more problematic as the true biological nature of different layers of chromatin organization is still not completely understood. Striking examples of this are the TADs, which can be identified using a large array of methodological solutions, but their structure and function is not completely understood yet. In particular, their internal structure remains elusive as beyond the resolution limit of Hi-C and also of super resolution microscopy techniques. Addressing both the biological and technological open challenges will allow achieving a complete understanding of the functional role of chromatin 3D architecture.
